# Pelvic Aneurysmal Bone Cyst in an Adolescent: A Case Report and Literature Review

**DOI:** 10.7759/cureus.9534

**Published:** 2020-08-03

**Authors:** Hadeel H Alalawi, Shoog Alfadhel, Mahbub Khan, Abdulrahman Bobseit

**Affiliations:** 1 Department of Orthopedics, King Fahad General Hospital, Madinah, SAU; 2 Orthopedics, College of Medicine, King Saud Bin Abdulaziz University for Health Science, Riyadh, SAU; 3 Department of Orthopedics, Prince Sultan Military Medical City, Riyadh, SAU

**Keywords:** aneurysmal bone cyst, selective trans-arterial embolization, pelvis, benign

## Abstract

An aneurysmal bone cyst (ABC) is a benign but locally aggressive lesion. The challenge in managing pelvic ABC arises from its relative inaccessibility and the presence of nearby neurovascular structures. In this report, we present the case of a 14-year-old female with pelvic ABC and describe the symptoms, signs, and radiographic appearance of the ABC, management, and good outcome of non-surgical management by selective trans-arterial embolization. Although challenging, non-surgical management of pelvic ABCs can result in a favorable outcome. In addition, we reviewed the literature regarding the treatment modalities of pelvic ABCs.

## Introduction

An aneurysmal bone cyst (ABC) is a rare benign, reactive, locally aggressive, and highly vascular tumor of unknown origin. It is an intraosseous and rare soft tissue lesion. It accounts for about one percent of benign bone tumors [1]. ABC can be a secondary to giant cell tumor, chondroblastoma, osteoblastoma, and osteosarcoma in 30% of cases [2]. In 52% of the cases, ABCs are found in the long bone metaphysis, as well as the sternum and spinal column [3].

ABC also occurs in the pelvis (8%-12%) [4]. Several factors make the management of pelvic ABC challenging, including difficulty in approaching the lesions, difficulty in achieving intraoperative hemostasis, nearby neurovascular bundles, and risk of injury to the acetabulum or sacroiliac joint that may affect the pelvic stability. Herein, we present a case of pelvic ABC, which treated successfully without complications via selective trans-arterial embolization (STAE). 

## Case presentation

A 14-year-old female patient, without remarkable medical conditions, presented to the clinic with left hip pain for one year. The pain was progressive and not relieved by medications. She was limping with no history of constitutional symptoms. Physical examination findings revealed antalgic gait and limited range of motion due to the pain with mild swelling.

**Figure 1:**
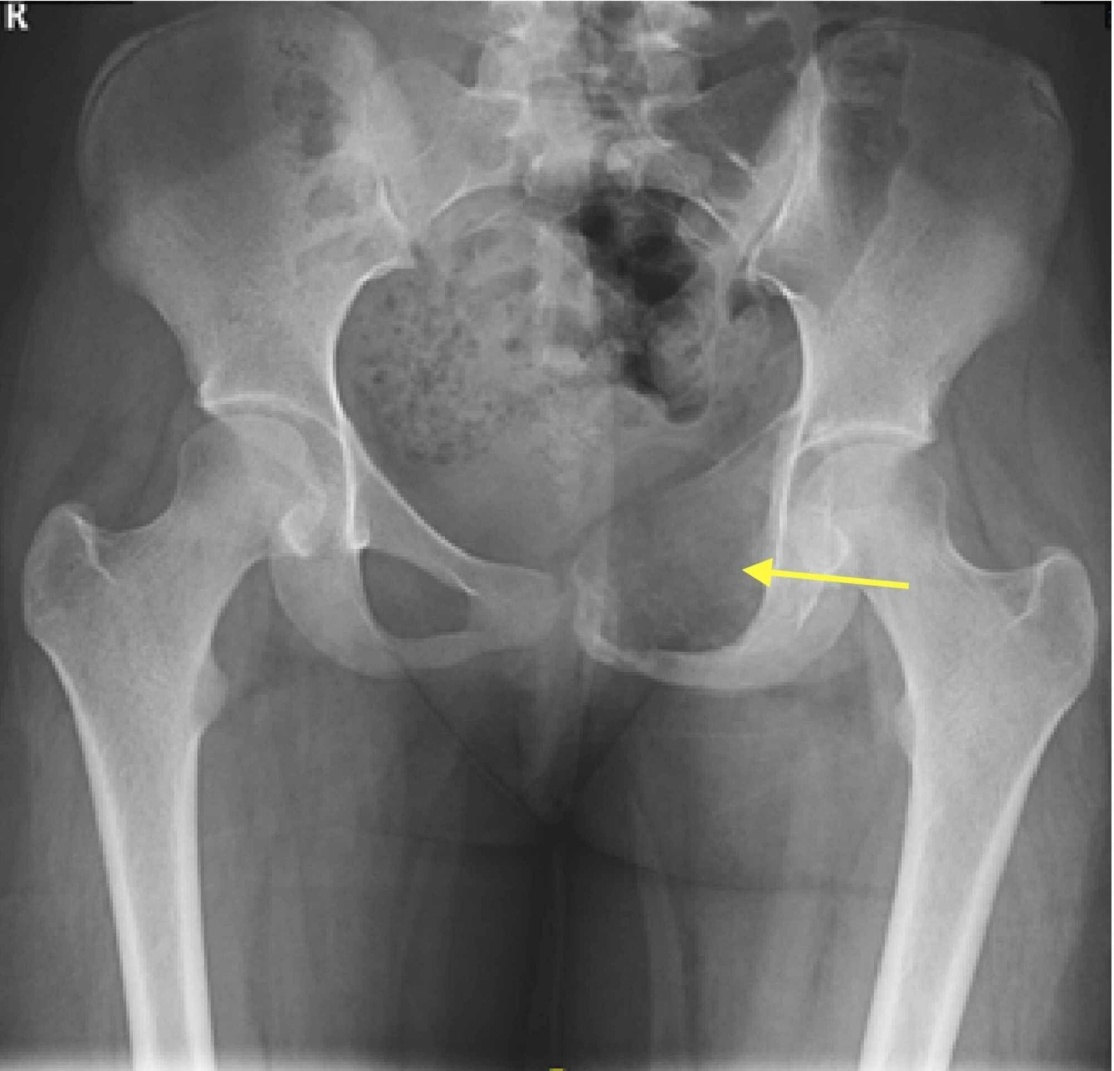
Anteroposterior radiograph of the pelvis Showing an expansile osteolytic lesion involving the left superior pubic ramus and extending to the anterior wall of the left acetabulum.

**Figure 2:**
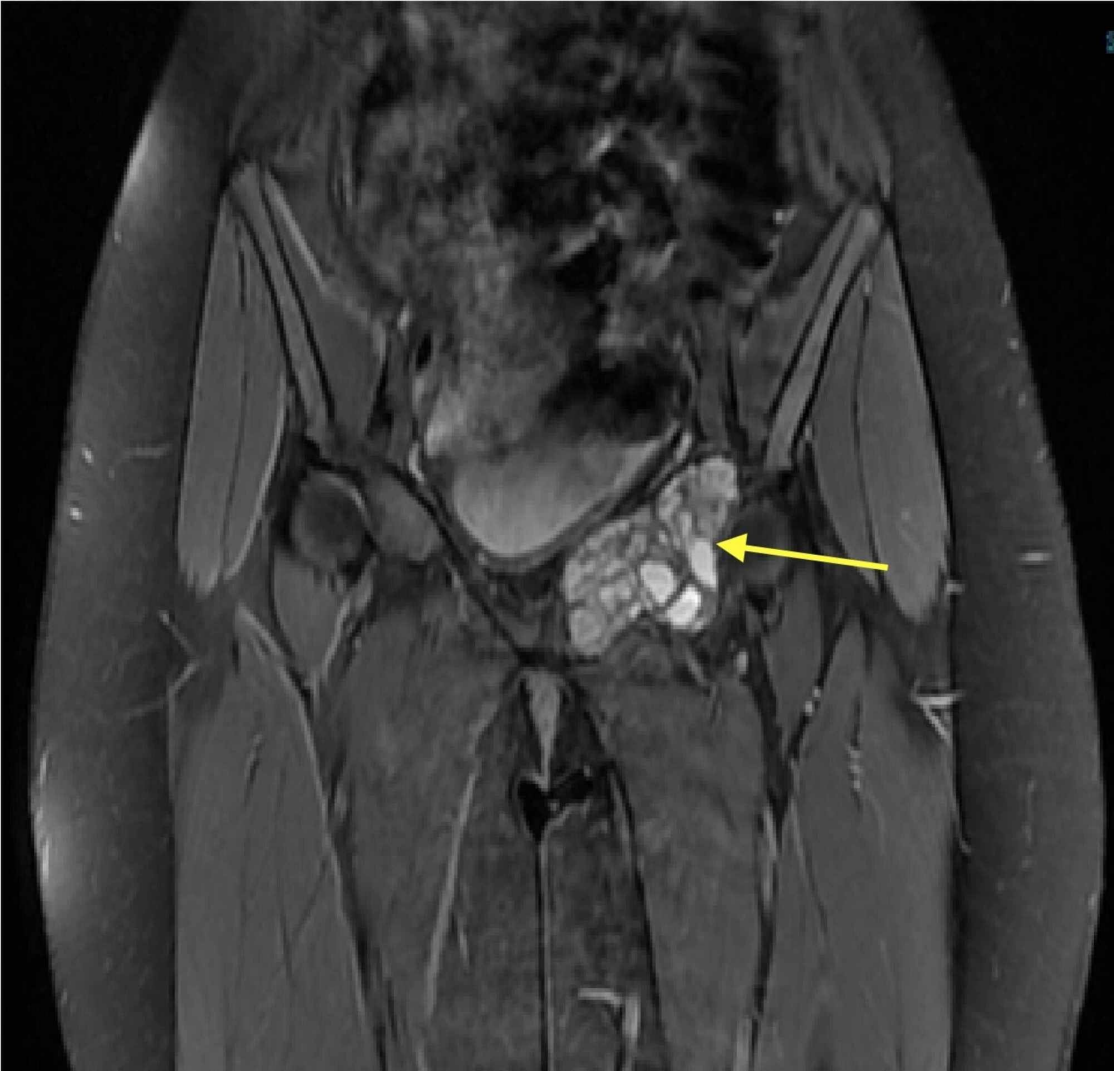
T2-weighted magnetic resonance image Coronal view showed 6×4.5×3.5 cm well-defined lesion and demonstrated internal septations forming cysts containing fluid-like signal intensity.

The pelvic radiographs showed an expansile osteolytic lesion involving the left superior pubic ramus and reaching to the left acetabulum anterior wall (Figure 1). Pelvic magnetic resonance imaging (MRI) showed fluid-fluid levels compatible with an aneurysmal bone cyst (ABC) (Figure 2). Tumor workup was done. Computed tomography-guided biopsy and histology revealed an ABC. 

The prognosis and treatment were discussed with the patient’s parent. Her parent consented for STAE. Under local anesthesia, angiography and selective arterial embolization were carried out via the left common femoral artery. Two feeding arteries were identified that originating from the inferior epigastric and circumflex arteries. The feeding vessels were selectively catheterized using a 5-French diagnostic catheter and a microcatheter. Embolic agent polyvinyl alcohol (PVA) was injected successfully. Thereafter, angiography through the internal iliac artery anterior division demonstrated complete and successful embolization of the arterial supply to the lesion. On the next day, the patient has discharged home pain-free and allowed full weight-bearing.

**Figure 3:**
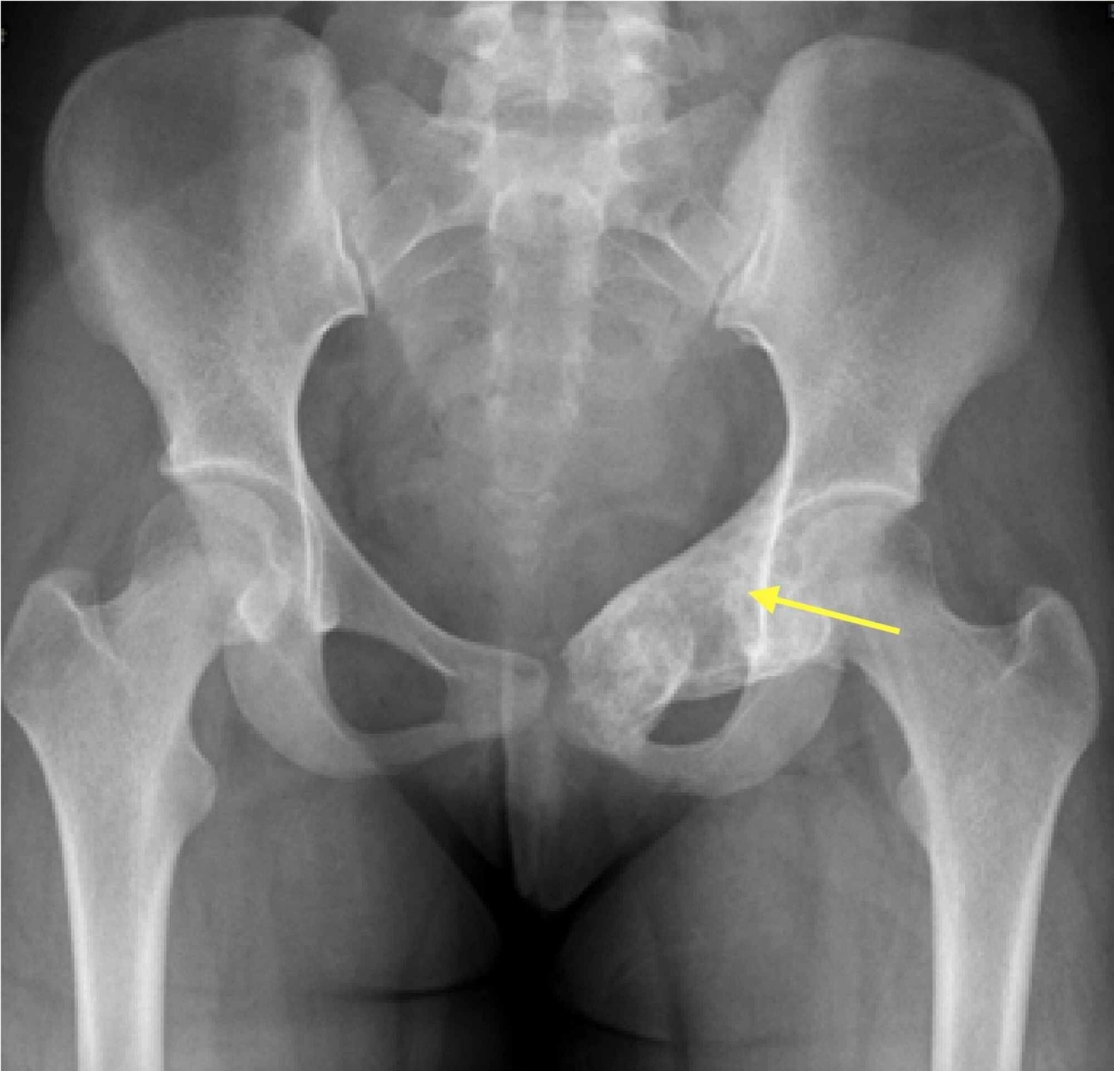
Anteroposterior radiograph of the pelvis four years post embolization Showing homogenous trabecular bone formation and ossification of the lesion.

**Figure 4:**
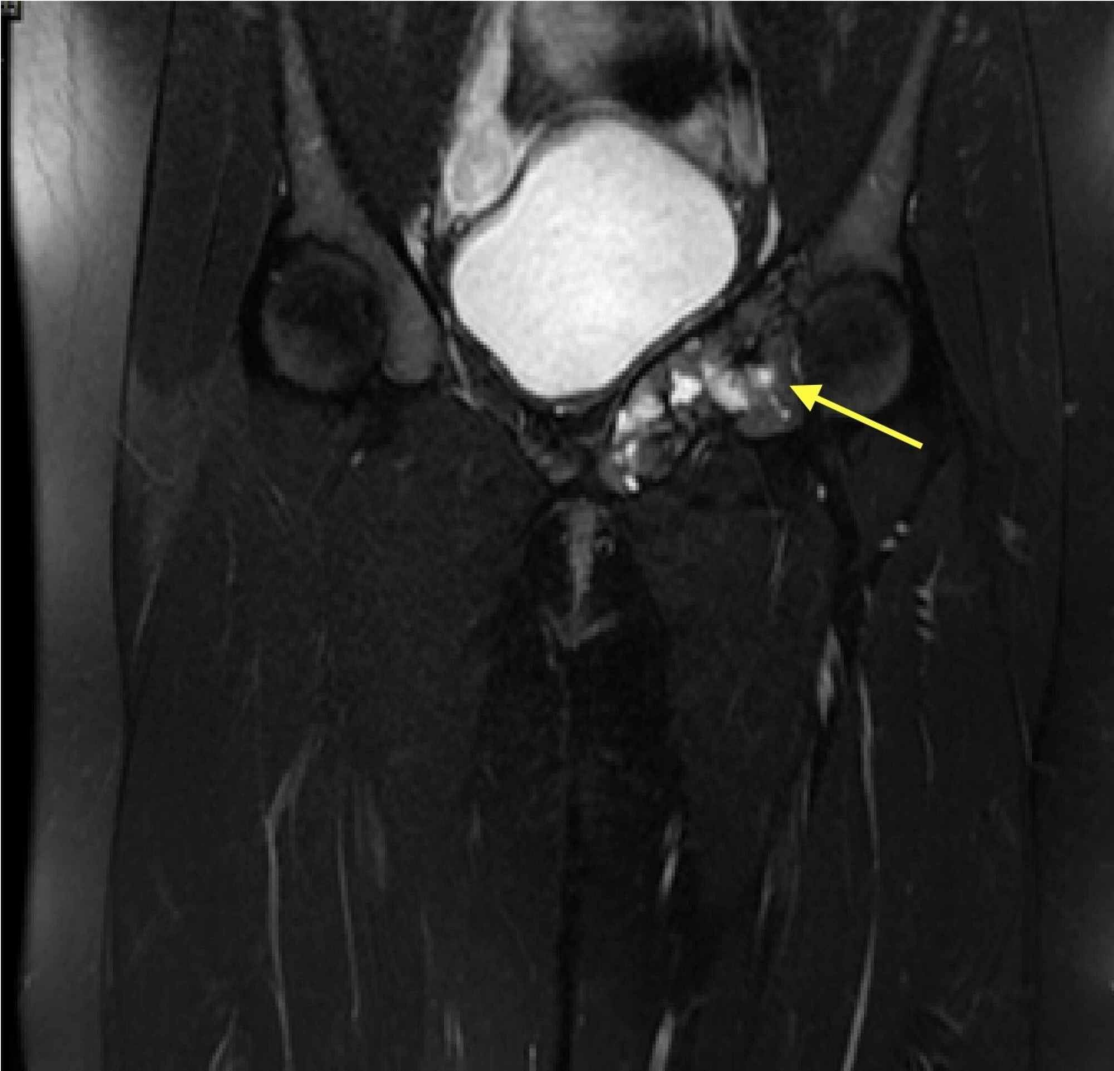
T2-weighted magnetic resonance image of the pelvis four years post embolization Showed a reduction in size as well as cystic appearance with no new bony lesion.

The patient had a routine follow-up evaluation every three months initially and at six months thereafter. Follow-up routine evaluation included radiographs of the pelvis and left hip joint. Radiographs revealed progressive trabecular bone formation and a gradual reduction of the size of the lesion (Figure 3). At four year follow-up, she was symptom-free and able to walk without limping. MRI showed a reduction in the size as well as a cystic appearance with no local recurrence (Figure 4). 

## Discussion

ABC is a benign bone lesion with multiple blood-filled cavities. It is highly associated with the destruction of the bone, fractures, and recurrence. It commonly occurs in patients aged <20 years [5]. For instance, our patient was 14 years old upon presentation. Pain is the most presenting symptom and may be associated with limping, restricted motion, and apparent mass.

Diagnosis is usually done by radiography and MRI [6]. In some cases, diagnosis can be missed due to misinterpreted symptoms, including pain that radiating to the medial or the anterior aspect of the thigh and performing knee rather than pelvic radiography [4]. Fluid levels on imaging are the most predictive of ABC.

Differential diagnoses of ABC included unicameral bone cysts, chondromyxoid fibroma, chondroblastoma, giant cell tumor, osteoblastoma, or telangiectatic osteosarcoma. Accurate diagnosis by histology is necessary because the prognosis and treatment of ABC are entirely different from those of other differential diagnoses [2]. In this case, MRI showed internal septations and the characteristic fluid-fluid levels. The biopsy was done to confirm the diagnosis and exclude other differential diagnoses.

The management of ABC most commonly consists of curettage and bone grafting, with or without adjuvant therapy. Other treatment modalities have been reported, including en bloc resection, sclerotherapy, radionuclide ablation, and selective arterial embolization. Management of pelvic ABC is challenging. By considering the patient’s age, location, size, vascularity, and degree of invasion of the lesions, the treatment should be carefully chosen.

In the present case, by considering the patient’s age, location of ABC, and risk of surgical treatment, the patient was treated by STAE. In most cases of pelvic ABC, STAE is performed preoperatively to decrease intraoperative bleeding for lesions with extensive soft-tissue expansion or lesions greater than 5-8 cm [4]. Also, it has been reported as a definitive treatment of ABC [7, 8]. There is no clinically significant difference between the embolic materials. The most important factor that determines the best choice of embolic material is operator experience [9]. In our report, PVA particles were used. The maximum number of embolization is seven [10]. Following STAE, the recurrence can occur if the patient's age is under 16 years or the lesion's size is larger than 5 cm, thus embolization can be repeated in these situations. Most of the recurrence occurs within one year of initial embolization [6]. In the present case, only one session was done, as follow-up radiography and MRI showed no new lesion or recurrence, and complete devascularization of an ABC was achieved. The success rate of embolization for ABC ranges from 75% to 94% with a recurrence rate from 39% to 44%. Complications included skin necrosis, and temporary paresis can occur in 5% of patients [8]. However, the application of STAE is limited by lesions with non-identifiable feeding vessels or maybe fed by vessels that supply nearby critical tissues and organs [11].

Other modalities included denosumab therapy and percutaneous treatment with calcitonin and methylprednisolone. Denosumab is a monoclonal antibody targeted against receptor activator of nuclear factor kappa-B ligand (RANKL), its use is dependent on RANK, and RANKL expression was observed histologically in ABC. Ntalos et al. report a case of primary pelvic ABC in a 35-year-old woman, which was not amenable to surgical intervention [12]. Denosumab led to a significant shrinkage of the lesion. Then, the patient underwent surgical intervention in addition to preoperative STAE. At six months, ABC recurred, which was treated only with denosumab, and a good clinical outcome was observed; however, more cases of pelvic ABC are still needed to further study to conclude its efficacy in the treatment of pelvic ABC [13]. Table 1 shows a summary of the published literature regarding the management of pelvic ABC [1, 4, 14-19].

**Table 1:** Published literature

Authors	Number of patients	Age	Site	Therapy	healed	Non-healed	Complications
Grahneisa F, et al. [14]	65	4 – 74	23% pelvis	Six - resected, five - minimal invasive, 54 - curettage	34	-	10 - persistence, 12 - recurrence
Palmerini E, et al. [15]	9	14 – 42	Six - pelvis	Denosumab + surgery	2	-	-
Papagelopoulos P, et al. [4]	40	2 – 44	12 - sacrum, seven - pubis, seven - ischium, seven - posterior column, seven -acetabulum	14 - intralesional curettage, 21 - excision curettage	40	-	10 - recurrence, two - tear in the dura matter, three - cut of the lower nerve roots, two - deep infections, one - bowl obstruction
Capanna R, et al. [16]	23	3 – 45	Four - ischium acetabulum, one - ischium, two - ileum acetabulum, one - ileum, 13 - ileopub ram acetabulum + neck of ileum, one - hemipelvis, one - ischiopub ram	Seven - curettage, four - curettage +homograft, three - resection, two - biopsy, six - biopsy + radiation therapy, one - curettage + radiation therapy	-	-	Three - recurrence, one - hypoesthesia, one - quadriceps and psoas deficit, one - crural nerve palsy, one - limb shortened 6.5 cm, fem head necrosis, one - ankylotic hip and deep wound infection
Cottalorda J et al. [1]	15	1.5 – 15	Five - iliopubic ramus, four - ischiopubic ramus, two - acetabulum with ischiopubic ramus, two - ilium, one - acetabulum with iliopubic ramus	Six - intralesional curettages, two - intralesional curettages + bone graft, three - selective arterial embolization (SAE), one - preoperative SAE and curettage, one - curettage and autogenous bone grafting, two - biopsies alone	-	One - large residual	Two - recurrence
Sobeai M, Juhani W [17]	1	24	Right hip	Surgical resection after embolization	1	-	-
Dubois J, et al. [18]	17	4 – 15	Six - extremities, two - pelvis, two - spine, five - mandible, one - rib, one - sphenoid bone	Sclerotherapy	17	-	Two - inflammatory reaction, one - leakage, one - small blister
Brosjö O, et al. [19]	38	3 – 26	One - pubic bone, two - sacrum, one - acetabulum	Percutaneous sclerotherapy	33	Two - residual pain, two - follow up for symptoms	Three - inflammatory reactions, one - hospital admission, two - required analgesia, one - moderate flexion contracture

## Conclusions

In treating ABC is essential to exclude other differential diagnoses by biopsy, as the management of ABC is entirely different. As mentioned in the literature review, in some cases, spontaneous healing was documented after a biopsy but most of them have been observed for a long time. Regarding operative management, en bloc resection is out of favor as such aggressive intervention does not appear to be indicated. Curettage and bone grafting is still the main way of management. As there is a risk of intro-operative bleeding, STAE before surgery is indicated. Although few cases were reported about the use of STAE as the definitive treatment of pelvic ABC, a good result was achieved in most of these cases. In addition to pre-operative STAE, denosumab has recently been used as a neoadjuvant for surgery and has a reasonable result when used alone to treat the recurrence. Since denosumab can contribute to shrinkage of the tumor, its use with STAE alone has not been reported before. In our case, we treated a primary pelvic ABC in an adolescent non-surgically by a single session of STAE with an excellent outcome. Even though the management of pelvic ABC is challenging, good results can be fulfilled with non-surgical treatment if the patient is selected and treated appropriately.
